# Serum TNF-a and biomarkers levels after tenofovir therapy in patients with serum HBeAg-positive chronic hepatitis B

**DOI:** 10.5937/jomb0-56238

**Published:** 2025-06-13

**Authors:** Xijie Lai, Guosheng Gao, Xiunong Jiang

**Affiliations:** 1 Ningbo No. 2 Hospital, Department of Liver Disease, Ningbo, Zhejiang Province, China; 2 Ningbo No. 2 Hospital, Department of Clinical Laboratory, Ningbo, Zhejiang Province, China

**Keywords:** chronic hepatitis B, tenofovir amibufenamide, entecavir, liver function, renal function, TNF-a, antiviral efficacy, inflammatory markers, hronični hepatitis B, tenofovir amibufenamid, entekavir, funkcija jetre, funkcija bubrega, TNF-a, antivirusna efikasnost, inflamatorni markeri

## Abstract

**Background:**

Chronic hepatitis B (CHB) remains a wide-spread and serious infectious disease, with its pathogenesis still not fully understood. Tenofovir Amibufenamide, a pro-drug of tenofovir, belongs to the class of nucleoside reverse transcriptase inhibitors and is used in the treatment of CHB. This study aimed to evaluate the efficacy and safety of Tenofovir Amibufenamide in treating HBeAg-positive CHB.

**Methods:**

A total of 60 patients diagnosed with HBeAg-positive CHB were randomly assigned to two groups: an entecavir (ETV) group (0.5 mg) and a Tenofovir Amibufenamide (TMF) group (0.25 mg), with 30 patients in each. After 24 months of treatment, renal function (serum creatinine, glomerular filtration rate), liver function (ALT , AST , total bilirubin), and inflammatory markers (TNF-a levels) were measured to assess the antiviral efficacy and safety profiles of the two treatments.

**Results:**

Patients in the TMF group exhibited smaller changes in serum creatinine and glomerular filtration rate, indicating less renal impairment compared to the ETV group. Furthermore, the TMF group demonstrated greater reductions in alanine aminotransferase (ALT) and total bilirubin (TBIL) levels, indicating superior improvement in liver function (P<0.05). TNF-a levels were significantly elevated in the ETV group, reflecting greater inflammatory activity, while the TMF group showed more controlled inflammation. Additionally, the TMF group experienced significantly lower hepatitis B surface antigen (HBsAg), hepatitis B e antigen (HBeAg), and HBV DNA levels, showing superior antiviral efficacy. The incidence of adverse reactions was lower in the TMF group (3.3%) compared to the ETV group (13.3%), although the difference was not statistically significant (P>0.05).

**Conclusions:**

Tenofovir Amibufenamide demonstrated superior efficacy in improving liver function and reducing viral load in patients with HBeAg-positive CHB. Moreover, it had a minimal impact on renal function and presented a higher safety profile compared to entecavir. These findings suggest Tenofovir Amibufenamide as a promising alternative for the treatment of HBeAg-positive CHB.

## Abstract (sr)

**Uvod:**

Hronični hepatitis B (CHB) i dalje predstavlja rasprostranjenu i ozbiljnu infektivnu bolest, čija patogeneza nije u potpunosti razjašnjena. Tenofovir amibufenamid, prekurzor tenofovira, pripada klasi inhibitora reverzne transkriptaze nukleozida i koristi se u lečenju CHB. Cilj ove studije bio je da se proceni efikasnost i bezbednost teno-fovir amibufenamida u lečenju HBeAg-pozitivnog CHB.

**Metode:**

Ukupno 60 pacijenata sa dijagnozom HBeAg-pozitivnog CHB nasumično je podeljeno u dve grupe: grupa koja je primala entekavir (ETV) u dozi od 0,5 mg i grupa koja je primala tenofovir amibufenamid (TMF) u dozi od 0,25 mg, sa po 30 pacijenata u svakoj. Nakon 24 meseca terapije, procenjeni su parametri bubrežne funkcije (serum-ski kreatinin, glomerularna filtracija), funkcije jetre (ALT, AST, ukupni bilirubin) i inflamatorni markeri (nivoi TNF-a) kako bi se uporedila antivirusna efikasnost i bezbednosni profili dve terapije.

**Rezultati:**

Pacijenti u TMF grupi imali su manje promene u nivoima serumskog kreatinina i glomerularnoj filtraciji, što ukazuje na manju bubrežnu disfunkciju u poređenju sa ETV grupom. Takođe, TMF grupa je pokazala veće smanjenje nivoa alanin aminotransferaze (ALT) i ukupnog biliru bina (TBIL), što ukazuje na superiorno poboljšanje funkcije jetre (P<0,05). Nivoi TNF-a bili su značajno povišeni u ETV grupi, što odražava veću inflamatornu aktivnost, dok je u TMF grupi upala bila bolje kontrolisana. Pored toga, u TMF grupi su zabeleženi značajno niži nivoi površinskog antigena hepatitisa B (HBsAg), antigena hepatitisa B e (HBeAg) i HBV DNK, što ukazuje na bolju antivirusnu efikasnost. Učestalost neželjenih reakcija bila je niža u TMF grupi (3,3%) u poređenju sa ETV grupom (13,3%), iako razlika nije bila statistički značajna (P>0,05).

**Zaključak:**

Tenofovir amibufenamid je pokazao superiornu efikasnost u poboljšanju funkcije jetre i smanjenju virusnog opterećenja kod pacijenata sa HBeAg-pozitivnim CHB. Takođe, imao je minimalan uticaj na bubrežnu funkciju i povoljniji bezbednosni profil u poređenju sa entekavirom. Ovi nalazi ukazuju na to da tenofovir amibufenamid predstavlja obećavajuću alternativu za lečenje HBeAg-pozitivnog CHB.

## Introduction

Chronic hepatitis B (CHB) is a widespread and significant infectious disease with a global prevalence, affecting over 250 million people worldwide. If left untreated, CHB can lead to severe liver complications, including cirrhosis, liver failure, and hepatocellular carcinoma [1]. The pathogenesis of CHB is complex, involving direct viral replication, immune dysregulation, and an inflammatory response that leads to liver damage [2] [3]. Chronic infection is often accompanied by heightened levels of hepatitis B virus (HBV) replication, marked by elevated serum HBV DNA, which further accelerates liver inflammation and fibrosis. Effective early intervention is essential to reduce complications, prevent disease progression, and improve long-term survival rates in CHB patients.

The main goal of CHB treatment is to suppress HBV replication to the lowest possible level, thus minimising liver damage and preventing the progression to cirrhosis, liver failure, and hepatocellular carcinoma. This is typically achieved through antiviral therapy aimed at reducing HBV DNA levels, normalising liver enzymes (such as alanine aminotransferase [ALT] and aspartate aminotransferase [AST]), and preventing immune-mediated liver injury [4] [5]. Common biochemical markers used to monitor disease progression and treatment efficacy include serum HBV DNA levels, hepatitis B surface antigen (HBsAg), hepatitis B e antigen (HBeAg), ALT, and AST. Additionally, liver function tests, such as total bilirubin (TBIL) and albumin, as well as renal markers like serum creatinine (Scr) and glomerular filtration rate (GFR), are routinely assessed to gauge treatment safety and organ function.

The mainstay of CHB therapy has traditionally involved the use of nucleoside analogs, including lamivudine, entecavir (ETV), and tenofovir, all of which effectively inhibit HBV replication and reduce liver inflammation [6]. However, while these agents can effectively control the viral load, they are unable to fully eradicate HBV, and long-term use can lead to the development of drug resistance [7]. Both entecavir and tenofovir have robust antiviral properties and a high genetic barrier to resistance, making them first-line therapeutic choices for CHB patients [6]. Nonetheless, the need for more effective and safer treatment options remains, particularly given the potential for long-term adverse effects on renal function and bone density with tenofovir disoproxil fumarate (TDF) [8].

Tenofovir Amibufenamide, a novel prodrug of tenofovir, has recently emerged as a promising alternative. Approved in June 2021, Tenofovir Amibu fenamide belongs to the class of nucleoside reverse transcriptase inhibitors. Unlike TDF, Tenofovir Amibufen a mide exhibits superior cellular membrane penetration, which allows for targeted liver cell delivery, thus enhancing its antiviral efficacy while reducing systemic exposure and associated side effects [9] [10]. Notably, studies have shown that a lower dosage of Tenofovir Amibufenamide (as little as 1/10th of TDF) can achieve similar antiviral effects, with less impact on bone density and kidney function [11].

Despite its promising profile, the clinical effectiveness and safety of Tenofovir Amibufenamide in treating HBeAg-positive CHB patients remains insufficiently explored. This study aims to evaluate and compare the clinical outcomes of Tenofovir Amibu fenamide with entecavir in patients with HBeAg-positive CHB, focusing on key markers such as HBV DNA, HBsAg, HBeAg, liver function (ALT, AST, TBIL), renal function (Scr, GFR), and tumor necrosis factor-alpha (TNF-α) as indicators of inflammation and immune response. The goal is to provide further insights into the potential of Tenofovir Amibufenamide as a treatment option, offering a safer and more effective alternative for patients with CHB.

## Materials and methods

### Baseline data

Sixty CHB patients treated at Ningbo No. 2 Hospital from January 2024 to May 2024 were selected in this work. This study was approved by the Ningbo No.2 Hospital Ethics Committee (PJ-KY-2017-070-01), and informed consent was obtained from all participants. Patients enrolled had to satisfy all the following conditions: (1) meeting the diagnostic criteria of the *2019 China CHB Prevention and Treatment Guidelines* and having not received any antiviral treatment in the past; (2) testing positive for HBeAg; and (3) Age 18 years. Patients with any of following conditions had to be excluded from this work: (1) cooinfection with HIV, HCV, HDV, or other human immunodeficiency viruses; (2) alcoholic hepatitis, drug-induced hepatitis, or autoimmune liver disease; (3) cirrhosis or abnormally elevated serum alpha-fetoprotein levels; (4) abnormal ALT or AST levels in the past year; (5) comorbid psychiatric disorders or other severe organ diseases; (6) pregnant or lactating women; and (7) use of antiviral or immunomodulatory drugs before admission.

Based on the treatment medication, the patients were grouped: the ETV group and the Amibufenamide (TMF) group, with n = 30. The ETV group enrolled 13 females and 17 males; 3 had an education level of primary school or below, 16 had a secondary school education, and 11 had a high school education or above; the mean age was (39.76±9.34) years; the mean duration of illness (DOI) was (5.24±2.18) years. The TMF group comprised of 11 females and 19 males; 2 had an education level of primary school or below, 19 had a secondary school education, and 9 had a high school education or above; the mean age was (40.00±9.15) years; the mean DOI was (5.33±1.96) years. Differences in general characteristics of patients in the ETV and TMF groups were not great (*P*>0.05).

### Treatment schemes

Patients in ETV group experienced oral treatment with entecavir (manufacturer: Shanghai Sine Promod Pharmaceutical Co., Ltd.; approval number: H20052237; specification: 0.5 mg * 7 tablets), 1 tablet per dose, once a day. Patients in the TMF group received oral treatment with Amibufenamide (Changzhou Hengbang Pharmaceutical Co., Ltd.; approval number: HH20210029; specification: 0.25 mg * 30 tablets), 1 tablet per dose, once a day. The treatment duration for both groups was 1 year, during which alcohol consumption and the use of other drugs with similar effects were prohibited.

### Observation parameters

Fasting venous blood samples were collected from patients at baseline (0 months), 12 months post-treatment (12 months), and 24 months posttreatment (24 months). Levels of hepatitis B surface antigen (HBsAg) and hepatitis B e antigen (HBeAg) were determined by chemiluminescence, with reagents provided by Abbott Diagnostics Products Co., Ltd. An automatic biochemistry analyser was used to detect levels of alanine aminotransferase (ALT), aspartate aminotransferase (AST), total cholesterol (TC), triglycerides (TG), total bilirubin (TBIL), and serum creatinine (Scr), with all reagents provided by Ningbo Purebio Biotechnology Co., Ltd. The levels of TC and TG were measured by enzymatic methods; TBIL was determined by a modified Jaffe method with diazo reagent; Scr levels were measured using enzymatic methods, and the glomerular filtration rate (eGFR) was calculated accordingly. Tumor necrosis factor-alpha (TNF-α) levels were detected by flow cytometry, with reagents supplied by Hangzhou Sijiqing Biological Engineering Materials Co., Ltd., while serum hepatitis B virus gene (HBV-DNA) levels were determined using fluorescent quantitative PCR, with reagents provided by Daan Gene Co., Ltd. of Sun Yat-sen University.

### Methods for statistical analysis

Data were statistically analysed using SPSS 19.0. Categorical data were presented as frequencies or rates, and the χ^2^ test was employed for intergroup comparisons. Continuous data were expressed as means±standard deviation, and the t-test was used for intergroup comparisons. *P*<0.05 was considered statistically significant for all analyses.

## Results

### Demographic data

A total of 60 patients with HBeAg-positive chronic hepatitis B (CHB) were enrolled in this study, with 30 patients assigned to the entecavir (ETV) group and 30 patients to the Tenofovir Amibufenamide (TMF) group. The baseline demographic characteristics of the two groups were similar, ensuring comparability between the groups.

In the ETV group, the mean age was 39.76±9.34 years, and the cohort consisted of 13 females and 17 males. The mean duration of illness (DOI) was 5.24±2.18 years. Among the participants, 3 had an education level of primary school or below, 16 had completed secondary school, and 11 had a high school education or higher.

The TMF group had a mean age of 40.00±9.15 years, with 11 females and 19 males. The mean DOI was 5.33±1.96 years. In terms of education level, 2 patients had primary school education or below, 19 had completed secondary school, and 9 had a high school education or higher. There were no significant differences in age, sex distribution, or DOI between the two groups (P>0.05), indicating comparable baseline characteristics.

These demographic factors were similar across both treatment groups, confirming that any observed differences in treatment outcomes were likely due to the interventions themselves.

### Changes in blood lipid levels in different groups

**Figure 1:**
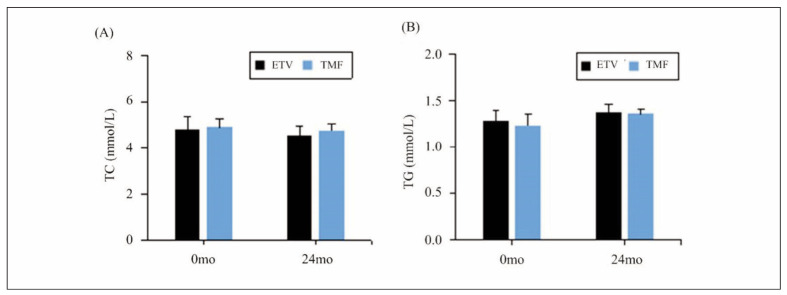
Changes in TC (A) and TG (B) levels before and after the patients were treated.

Changes in TC and TG indicators before and after treatment were analysed for both the ETV and TMF group, as explicated in Figure 1. No remarkable differences were visualised in these indicators between two groups before the patients were intervened (*P*>0.05). Additionally, the post-treatment TC and TG levels in serum of patients between the ETV and TMF group exhibited no considerable differences (*P*>0.05).

### Changes in renal function

**Figure 2:**
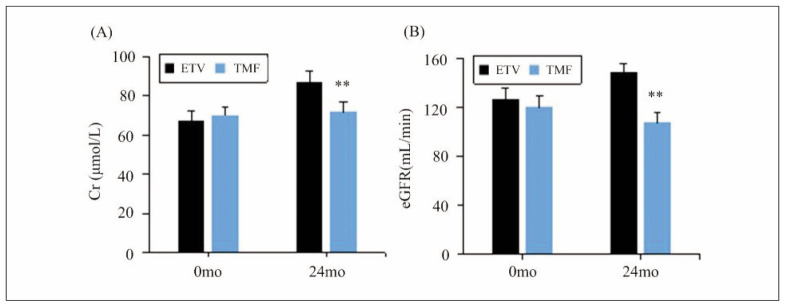
Changes in renal function-related indicators.

The changes in levels of Cr and eGFR indicators before and after treatment were analysed for both the ETV group and the TMF group, as demonstrated in Figure 2. Differences in serum Cr and eGFR levels were observed to be small for patients between the ETV group and the TMF group before they were treated (*P*>0.05). After treatment, the ETV group exhibited a remarkable variation in Cr and eGFR levels, which were much changes in contrast to those in the TMF group, showing obvious differences (*P*<0.05).

### Changes in liver function of patients before and after different treatments

**Figure 3:**
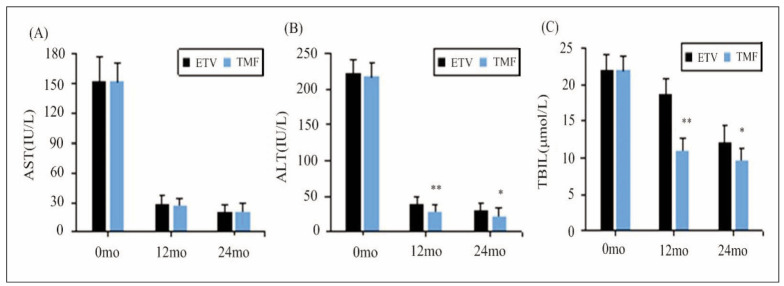
Changes in liver function of patients before and after different treatments.<br>((A): AST; (B): ALT; and (C): TBIL).<br>Note: ** suggested a great difference with P<0.01 to the ETV group

Figure 3 below summarises the changes in AST, ALT, and TBIL indicators before and after treatment. They all showed no great changes in serum of patients in different groups before treatment (*P*>0.05). After 12 months and 24 months of treatment, all patients showed varying degrees of reduction in AST, ALT, and TBIL levels, with the TMF group exhibiting much lower ALT and TBIL, demonstrating visible differences compared to the ETV group (*P*<0.05).

### Changes in TNF-α level of patients

**Figure 4:**
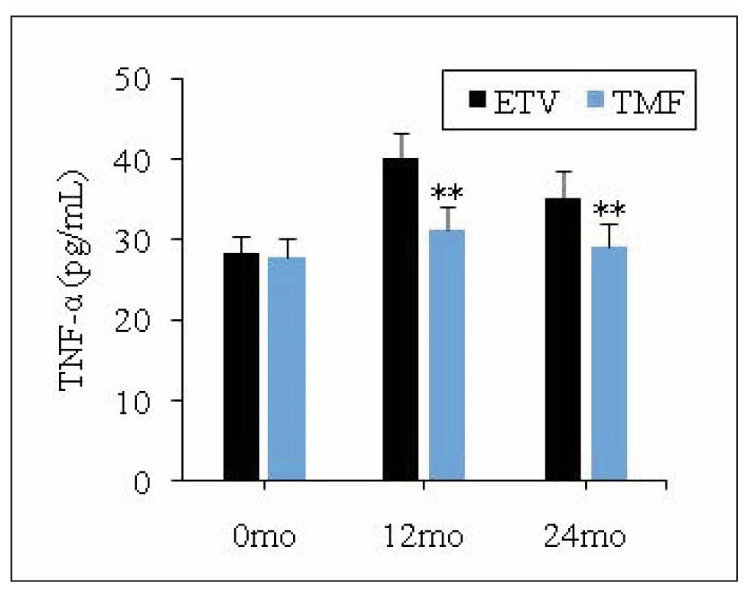
Variation in TNF-α level of patients.<br>Note: * and ** suggested a great difference with P<0.05 and P<0.01 to the ETV group, respectively

Figure 4 explicated the changes in TNF-α before and after treatment for patients in different group. As it detailed, TNF-α level was not greatly different for patients in ETV group and TMF group before the patients were treated (*P*>0.05). After 12 months and 24 months of treatment, all patients experienced varying degrees of increase in TNF-α levels, with a more pronounced increase in ETV group. Notably, cases in TMF group exhibited obviously lower TNF-α based on those in ETV group, demonstrating a remarkable difference (*P*<0.05).

### Comparison of antiviral efficacy in two groups

**Figure 5:**
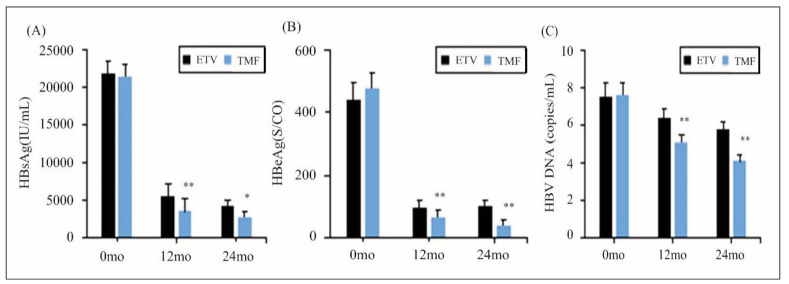
Variation of HBV-associated antigens and DNA.<br>((A): HBsAg; (B): HBeAg; and (C): HBV DNA).<br>Note: * and ** suggested a great difference with P<0.05 and P<0.01 to ETV group, respectively

Surely, the HBsAg, HBeAg, and HBV DNA would change before and after the patients were treated, and the details were detailed in Figure 5. It revealed no visible differences in all above parameters between patients in different groups before treatment (*P*>0.05). After 12 months and 24 months of treatment, all patients experienced varying degrees of reduction in HBsAg, HBeAg, and HBV DNA levels, with the TMF group exhibiting more obvious lower HBsAg, HBeAg, and HBV DNA, which presented considerable differences in contrast to the levels in ETV group (*P*<0.05).

### Comparison on IoAR

Differences in adverse reactions between the ETV group and the TMF group were compared in Table 1. After treatment, the ETV group had 3 cases of nausea and 1 case of fatigue as adverse reactions, with a total IoAR of 13.3%. In contrast, the TMF group had only 1 case of nausea as an adverse reaction, resulting a total IoAR of 3.3%. Thus, the corresponding IoAR in TMF group was lower in comparison to that in ETV group, but exhibited no great differences (*P*>0.05).

**Table 1:** IoAR in patients after different treatments [n (%)].

Group	Nausea	Fatigue	Total number of patients with adverse reactions
ETV group (n = 30)	3 (10.0)	1 (3.3)	4 (13.3)
TMF group (n = 30)	1 (3.3)	0 (0.0)	1 (3.3)
χ^2^ value			0.161
P value			0.353

## Discussion

Our results demonstrate that Tenofovir Amibufenamide (TMF) significantly improves liver and renal function in patients with HBeAg-positive CHB when compared to entecavir (ETV). The analysis of serum biomarkers reveals important mechanisms underlying these improvements. Specifically, TMF treatment resulted in a greater reduction in liver enzymes such as ALT and TBIL, indicating improved liver function. Additionally, serum TNF-a levels, a marker of inflammation, were lower in the TMF group, suggesting that TMF may reduce the inflammatory response associated with HBV infection. This reduction in inflammation could contribute to the prevention of liver cell damage and fibrosis. Moreover, the levels of HBsAg, HBeAg, and HBV DNA were significantly decreased in the TMF group, highlighting its potent antiviral effect and its ability to suppress viral replication more effectively than ETV. These findings support the notion that TMF not only suppresses HBV replication but also modulates immune responses and inflammation, which could lead to better long-term outcomes for patients with HBeAg-positive CHB.

CHB is a common infectious disease seen in clinical practice. HBeAg-positive represents a specific type of HBV infection within CHB patients. Compared to ordinary infected individuals, HBeAg-positive CHB patients exhibit higher viral replication activity and are in a persistent infection state, often characterised by elevated levels of HBV DNA [12] [13]. Furthermore, HBeAg-positive CHB patients are at a significantly increased risk of severe complications such as cirrhosis, liver function failure, and liver cancer [14]. Suppression of viral replication to the maximum extent possible can effectively slow down the progression of liver disease. Antiviral therapy is capable of inhibiting or interrupting HBV DNA replication and propagation, thus reducing damage inflicted by the virus on liver cells and ultimately achieving the goal of controlling viral transmission [15] [16] [17]. Entecavir and tenofovir are the most frequently applied antiviral drugs to treat CHB in clinical practice. However, the 5-year resistance rate for entecavir in CHB patients is 1.2%, while the resistance rate for tenofovir is 0.0% [18]. Tenofovir Amibufenamide can be activated by phosphorylation and effectively inhibits HBV DNA replication and progression, slows down liver fibrosis, and improves the survival of damaged liver cells [19]. Tenofovir Amibufenamide, a new second-generation derivative of tenofovir, is also the first original oral anti-HBV drug developed in China. Compared to teno fovir, Tenofovir Amibufenamide demonstrates greater effectiveness in reducing or clearing viral load in HBeAg-positive/negative CHB patients [8]. Its once-daily dosing regimen is more convenient and it shows advantages in lower resistance development.

In this work, efficacy and safety of Tenofovir Amibufenamide in treating HBeAg-positive CHB were investigated. Previous research has shown that HBeAg-positive CHB patients often exhibit varying degrees of liver function impairment, primarily indicated by elevated ALT and AST in serum [20]. Existing studies have confirmed that entecavir can effectively reduce liver cell damage, slow down progression of liver fibrosis and cirrhosis, and enhance patient liver function [21]. This work revealed that in contrast to the entecavir group, patients in Tenofovir Amibufenamide group exhibited significant reductions in ALT and TBIL levels after treatment. Previous research has indicated that a small number of HBV patients undergoing entecavir treatment may experience transient liver function abnormalities, which return to normal after discontinuation [22]. Besides, this work suggested that Tenofovir Amibufenamide can effectively enhance the liver function of patients suffering from HBeAg-positive CHB. Furthermore, the study also revealed that compared to the entecavir group, patients in the Tenofovir Amibufenamide group exhibited smaller differences in Cr and eGFR levels after treatment. The kidneys are the primary organs for drug metabolism, and several studies have shown that entecavir has a good safety profile in patients with mild impairment in renal function [23] [24] [25]. Notably, long-term administration of entecavir may trigger tubular dysfunction in patients (e.g., reduced urinary glucose and uric acid excretion) and other kidney-related issues [26]. In addition, this work indicated that compared to entecavir, Tenofovir Amibufenamide has a smaller impact on renal function in CHB patients and does not cause kidney damage.

HBeAg-positive CHB patients exhibit an abnormally activated immune system, accompanied by inflammatory reactions and immune cell infiltration. The degree of liver cell damage is closely related to the inflammatory response, leading to liver tissue fibrosis [27]. In recent years, research has shown that Th1/Th2 cells regulate the process of virus clearance, and an imbalance in Th1/Th2 cells greatly contribute to the chronicity of HBV infection [28] [29]. Th2 cells generate cytokines such as TNF-α, which regulate cellular immunity. TNF-α becomes activated and released during the inflammatory process. The degree of liver inflammation, liver damage, and liver fibrosis in CHB patients is positively linked with higher levels of TNF-α [30] [31] [32]. Moreover, TNF-α levels are also associated with the activity of HBV replication in CHB patients [33]. This study found that compared to the entecavir group, patients receiving Tenofovir Amibufenamide treatment exhibited a lower degree of elevation in serum TNF-a levels after treatment. This suggests that Tenofovir Amibufenamide may reduce inflammation caused by viral infection, thus preventing further damage to liver cells.

Prolonged HBV infection can contribute to apoptosis and damage of liver cells, affecting both the structure and function of the liver in patients [34]. The clearance of the HBV by the host immune system is crucial for reducing levels of HBsAg, HBeAg, and HBV DNA. For patients suffering from CHB, T cells and natural killer cells can specifically recognise and clear infected liver cells, thereby eliminating viral antigens (HBsAg and HBeAg) and reducing viral replication [35]. Lowered HBeAg and HBV DNA levels often accompanies transition of HBeAg, marking the shift of the virus from a highly replicative stage to a low replicative stage [36]. This transition is related to the extent of liver cell damage [37]. This work found that in contrast to the entecavir group, patients after the Tenofovir Amibufenamide intervention exhibited a remarkable decrease in levels of HBsAg, HBeAg, and HBV DNA in serum. This suggests that Tenofovir Amibufenamide directly suppresses HBV replication, thereby reducing HBV DNA levels. As the treatment progresses, viral load in patients with HBeAg-positive CHB decreases significantly, leading to reductions in HBsAg and HBeAg levels [38]. Lastly, this work asses sed the probability of adverse reactions occurring during the treatment period and found that 4 patients in the entecavir group experienced fatigue and nausea as adverse reactions, while only 1 patient in the Tenofovir Amibufenamide group experienced nausea. This indicates that Tenofovir Amibufenamide treatment for HBeAg-positive CHB has a higher level of safety.

## Conclusion

Both entecavir and Tenofovir Amibufenamide demonstrated effective therapeutic outcomes in interventing HBeAg-positive CHB. Among them, Tenofovir Amibufenamide exhibited a lower impact on renal function of these patients, and it was more effective in improving liver function, antiviral efficacy, and safety. This work was subjected to several limitations, including a small number of enrolled cases and a lack of analysis of factors influencing virological response. However, this work confirmed the clinical utility of Tenofovir Amibufenamide in treating HBeAg-positive CHB, highlighting its superior therapeutic effects and safety. These findings warranted further clinical application and promotion.

## Dodatak

### Funding

This study was supported by the Zhejiang Province and Ningbo City Co-constructed Project of Leading Medical & Health Discipline (Grant No. 2016-S04).

### Informed consent statement

The study is a retrospective study that will maximise the protection of the rights and privacy of the study participants and the content of the study. The results do not involve personal privacy and commercial interests and are exempt from informed consent.

### Ethics approval

The ethical considerations pertaining to this research have been rigorously examined and approved by the Ethics Committee of Ningbo 2 Hospital. The study involving the data adheres to the highest standards of ethical conduct and patient confidentiality. The approval from the Ethics Committee underscores our commitment to upholding the welfare and rights of all individuals involved in this study.

### Conflict of interest statement

All the authors declare that they have no conflict of interest in this work.
